# Structural analysis of *Brucella abortus* RicA substitutions that do not impair interaction with human Rab2 GTPase

**DOI:** 10.1186/1471-2091-13-16

**Published:** 2012-08-14

**Authors:** Bernard Nkengfac, Jenny Pouyez, Emilie Bauwens, Jean Vandenhaute, Jean-Jacques Letesson, Johan Wouters, Xavier De Bolle

**Affiliations:** 1 Molecular Biology Research Unit (URBM), Narilis, University of Namur, 61 Rue Bruxelles, Namur, B-5000, Belgium; 2 Laboratory of Structural Biological Chemistry (CBS), member of Narilis, University of Namur, 61 Rue Bruxelles, Namur, B-5000, Belgium; 3 Current address: McGill University, McGill University Health Centre, Royal Victoria Hospital, 687 Pine Ave. W., Room L3.05, Montreal, H3A 1A1, Canada

**Keywords:** Protein-protein interaction, Yeast two-hybrid, Mutagenesis, *Brucella*

## Abstract

**Background:**

Protein-protein interactions are at the basis of many cellular processes, and they are also involved in the interaction between pathogens and their host(s). Many intracellular pathogenic bacteria translocate proteins called effectors into the cytoplasm of the infected host cell, and these effectors can interact with one or several host protein(s). An effector named RicA was recently reported in *Brucella abortus* to specifically interact with human Rab2 and to affect intracellular trafficking of this pathogen.

**Results:**

In order to identify regions of the RicA protein involved in the interaction with Rab2, RicA was subjected to extensive random mutagenesis using error prone polymerase chain reaction. The resulting allele library was selected by the yeast two-hybrid assay for Rab2-interacting clones that were isolated and sequenced, following the “absence of interference” approach. A tridimensional model of RicA structure was used to position the substitutions that did not affect RicA-Rab2 interaction, giving a “negative image” of the putative interaction region. Since RicA is a bacterial conserved protein, RicA homologs were also tested against Rab2 in a yeast two-hybrid assay, and the *C. crescentus* homolog of RicA was found to interact with human Rab2. Analysis of the RicA structural model suggested that regions involved in the folding of the “beta helix” or an exposed loop with the IGFP sequence could also be involved in the interaction with Rab2. Extensive mutagenesis of the IGFP loop suggested that loss of interaction with Rab2 was correlated with insolubility of the mutated RicA, showing that “absence of interference” approach also generates surfaces that could be necessary for folding.

**Conclusion:**

Extensive analysis of substitutions in RicA unveiled two structural elements on the surface of RicA, the most exposed β-sheet and the IGFP loop, which could be involved in the interaction with Rab2 and protein folding. Our analysis of mutants in the IGFP loop suggests that, at least for some mono-domain proteins such as RicA, protein interaction analysis using allele libraries could be complicated by the dual effect of many substitutions affecting both folding and protein-protein interaction.

## Background

*Brucella abortus* is a facultative intracellular pathogen responsible for a worldwide zoonosis [1]. Like other intracellular bacteria such as *Legionella spp*[2-4] and *Salmonella spp*[5]*, B. abortus* probably depends on precisely orchestrated interactions with host cell proteins for its infectious process. Remarkably, these intracellular pathogens secrete proteins regulating host small GTPases [4-7]. Small GTPases of the Ras super family are signaling proteins that cycle between a GDP-bound inactive state and a GTP-bound active state. These two states are regulated by guanine-nucleotide exchange factors, which facilitate the conversion of GDP to GTP; GTPase activating proteins, which facilitate the hydrolysis of the GTP and Guanine-nucleotide-dissociation inhibitors, which negatively regulate the exchange activity of the GTPase and dislocate them from membranes. Rab GTPases are small GTPases playing a critical role in the control of membrane trafficking. Specifically, Rab2 has been shown to control membrane trafficking between the Golgi apparatus and the endoplasmic reticulum [8], Rab2 was also putatively associated with the phagosome [9] but without any known function in phagosomal maturation in mammalian cells [10]. RicA is an effector recently identified in *B. abortus*, which interacts with human Rab2 [6]. This interaction was detected using yeast 2-hybrid (Y2H) and confirmed by GST-pulldown. RicA has a preference for GDP-bound GST-Rab2 compared to GTPγS-bound GST-Rab2 [6]. Active Rab2 is known to be required for *B. abortus* intracellular proliferation [11]. A *B. abortus ΔricA* strain recruits less Rab2 on the *Brucella* containing vacuole, suggesting that RicA is playing a role during the intracellular trafficking of the bacterium [6].

RicA is predicted to belong to the superfamily of LβH proteins, comprising acetyltransferases, acyltransferases, carbonic anhydrases, ferripyochelin binding proteins, as well as many proteins of unknown functions. Their structure is characterized by the assembly of three β sheets in a left-handed “β helix” structure. In this paper, we attempted to localize the Rab2 interaction surface on the RicA predicted structure. We performed the “absence of interference” approach [12] previously used to map the interface of the catalytic domain of the DNA methylase Dnmt3a and its regulatory factor Dnmt3L. Mapping of the substitutions that do not disrupt the RicA-Rab2 interaction, on the predicted model of RicA structure, revealed two possible interfaces, a beta sheet and a loop called IGFP. The data reported here suggested that, of these two structural elements, at least the IGFP loop is also involved in RicA folding.

## Results

### Prediction of RicA three-dimensional structure

**Figure 1:**
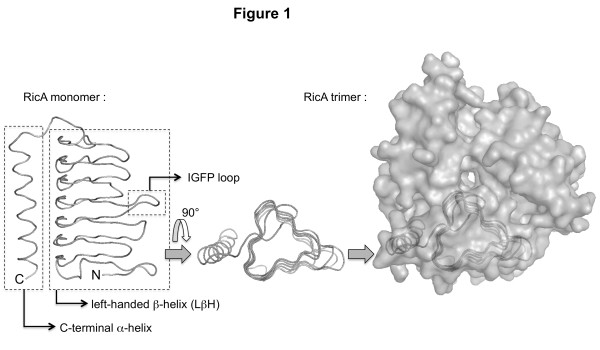
**Predicted three-dimensional structure of RicA.** The left-handed β-helix is composed of three β sheets connected by short loops. One of these loops is the IGFP loop (Ile69 to Pro73). The structure is shown as a ribbon aligned to the backbone of the model (made using the MacPyMol program), comprising residues Ile3 to Arg170. A “top view” is shown in the middle part of the figure. The three β sheets form a triangle in this view. On the right part of the figure, the accessible surface of the predicted trimer is depicted, with a monomer ribbon shown by transparency.

A His_6_ tagged version of RicA (His_6_-RicA) was overproduced, purified to homogeinity and tested in several crystallization protocols that failed (data not shown). We therefore modelized the RicA structure by homology and verified for model correctness using EsyPred3D [13] and verify3D [14] programs respectively. The three-dimensional (3D) structure of *Bacillus cereus* BC4754 sequence (1XHD code in protein databank, 41.9% identity) was used as the template for the homology modeling. The function of this *B. cereus* protein is unknown. Modeling using other templates (2EG0, 1V3W and 1THJ codes in protein databank) generated very similar models (data not shown). Conserved domain analysis of amino acid sequences of RicA and 1XHD revealed tandemly-repeating hexapeptide repeats (Hex-motif; [LIV]-[GAED]-X-X-[STAV]-X), indicating that the overall conformation of RicA contains a left-handed β-helical component (LβH) characteristic of acetyltransferases superfamily [15]. The RicA monomers were assembled as trimers. Indeed several homologs are trimeric, and the three histidine residues involved in zinc binding between the monomers, in the structure of carbonic anhydrase from *Methanosarcina thermophila* (1THJ code in protein databank), are conserved in RicA (His67, His84 and His89), suggesting that the trimeric structure is conserved in RicA. The predicted structure of RicA is presented in Figure 1. 

### RicA mutagenesis and selection of alleles allowing interaction with Rab2

RicA specifically interacted with Rab2 in the Y2H system as indicated by the induction of the *HIS3* (cell growth on plates lacking histidine in the presence of 3-aminotriazole, 3AT) and the *lacZ* (blue color when assayed with 5-bromo-4-chloro-3-indolyl-β-D-galactopyranoside) reporter genes (data not shown). In this interaction assay, RicA was fused with the transactivation coding sequence (AD) of Gal4p. Since fusion of RicA with the DNA binding domain (DB) of Gal4p was autoactivating the *HIS3* and *lacZ* reporters, the simple selection of ‘edgetic’ (interaction defective) alleles [16] was not possible. We therefore decided to use the previously proposed “absence of interference” approach [12], in which a possible interface is mapped on a three-dimensional (3D) structure thanks to the absence of interaction-disruptive substitutions in this region of the protein. Mutated RicA (525 bp) was synthesized by error-prone polymerase chain reaction (PCR) [17] from the pDEST-AD-RicA expression clone. A mutant library of about 10,000 clones was prepared in *E. coli*, by BP recombinational cloning of the PCR products in the pDONR201 vector. Five independent clones randomly selected were sequenced to check the mutation load. We found 36 mutations for the 2625 sequenced bases, i.e. a mutation frequency of 1.4%. The mutant library was transferred to the pDEST-AD vector and assayed with Rab2 in the Y2H to assess the influence of the mutations introduced into RicA. Among 1200 yeast clones, only 32 were positive for *lacZ* and *HIS3* reporters, indicating that only approximately 3% were able to interact with wild-type Rab2. A screening made with a slightly higher mutation rate for RicA coding sequence did not yield any positive interaction (data not shown), suggesting that 1.4% is close to the maximum mutation rate still allowing the recovery of interacting proteins, for this experimental setting. The *ricA* coding sequence was amplified from the 32 interacting clones by PCR and sequenced. We observed that a selective pressure occurred as the mutational load of 1.4% in the unselected library decreased to 0.5% in the selected clones. Among the 32 interacting clones, only two had the wild-type sequence, and all substitutions observed in other clones are reported in Table 1. A total of 29 substitutions were collected. As expected, some mutations are found in several clones, consistent with the hypothesis of their generation at different stages of the mutagenic PCR. For example, clones 5 and 10 are very similar, with four common substitutions and one additional substitution in clone 10. 

**Table 1:** Distribution of substitutions in the mutated RicA clones interacting with Rab2 in a Y2H assay

**Clone n°**	**Substitutions (and their score in the Blosum62 matrix)**
1, 12	T97S (1)
2	G8E (−2), G38S (0), D148V (−3)
3	N132Y (−2), R144C (−3)
4	V151I (3)
5	P2S (−1), D24H (−1), M101T (−1), V135A (0)
6	M127T (−1), E163D (2)
7	I65T (−1)
8	T121A (0)
9	I99N (−3), H166R (0)
10	P2S (−1), D24H (−1), I76N (−3), M101T (−1), V135A (0)
11	K10M (−1), P51T (−1)
13	M127V (−1)
14	E17G (−2), K124E (1)
15	I21V (3), E128V (−2), L143Q (−2)
16	H166R (0)
17	F13N (−3)
18	F39V (−1)
19	S157T (1)
20	M66L (2)
21	L45M (2), A119V (0)
22	I129V (3)
23	N7Y (−2)
24	S167T (1)
25	K30N (0)
26	Q12R (1)
28	K10E (1), M172L (2)
29	P2Q (−1)
31	T92S (1), A150T (0), G171C (−3)
32	I87T (−1)

Clones 27 and 30 did not contain substitutions. Clones 1 and 12 contained the same substitutions.'

### Rab2 interaction assay with RicA homologs

Since *B. abortus* RicA is conserved in many other bacteria, we tested the interaction of RicA homologs with human Rab2, using Y2H. Interestingly, by fusing *Caulobacter crescentus* RicA homolog to the AD of Gal4p, we detected interaction with human Rab2 in a Y2H assay using the *HIS3* and *URA3* reporters. The *C. crescentus* RicA homolog is sharing 52% identities with *B. abortus* RicA, indicating that it has a similar fold but with many substitutions, strongly suggesting that only conserved residues contribute to the interaction between RicA and Rab2.

### Structural analysis of the substitutions that do not impair RicA-Rab2 interaction

**Figure 2:**
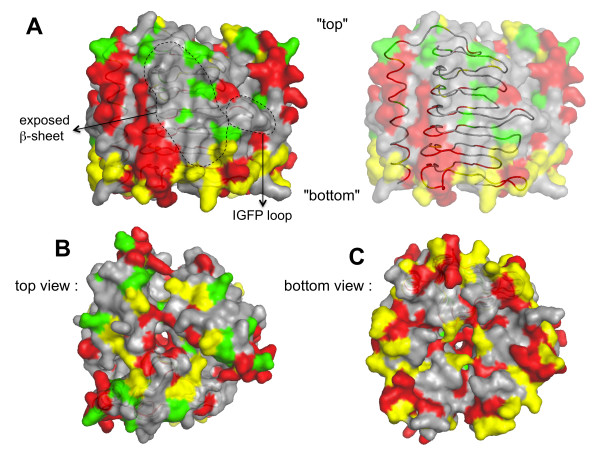
**Position of the LS substitutions that do not prevent RicA binding to Rab2.** The predicted accessible surface of the trimeric structure is depicted in each view. The position of the LS substitutions (score > 1 in the Blosum62 matrix) is indicated with the following color code : the substitutions found in mutated alleles only are shown in green, substitutions found in *C. crescentus* homolog only are shown in red, residues substituted in the mutated alleles and in the *C. crescentus* homolog are shown in yellow. In each picture, the ribbon of one monomer (as depicted in Figure 1) is shown by transparency, but in (A) on the right, the transparency is increased to allow the correspondence with Figure 1. (**A**) Position of the exposed β-sheet and the IGFP loop, in a “side view”. The arbitrary “top” and “bottom” of the structure are indicated. (**B**) and (**C**) Top and bottom views of the substitutions on the predicted trimeric structure of RicA, respectively.

**Figure 3:**
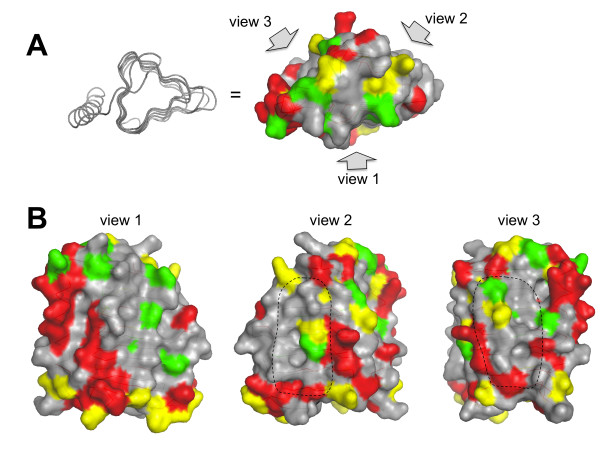
**Position of the LS substitutions on the monomeric model of RicA.** The structure is presented in the same orientation as in the middle pannel of Figure 1. Three possible views are possible. The view 1 is corresponding to the exposed face of the RicA monomer in the RicA tetramer. In views 2 and 3, the dashed line surrounds the region predicted to be involved in trimer formation. These regions are poor in LS substitutions, which is consistent with a trimeric structure preserved in the yeast two-hybrid assay and in the *C. crescentus* RicA homolog. As in Figure 2, the substitutions found in mutated alleles only are shown in green, substitutions found in *C. crescentus* homolog only are shown in red, residues substituted in the mutated alleles and in the *C. crescentus* homolog are shown in yellow.

The substitutions that do not impair mutated RicA binding to Rab2 in the Y2H assay were positioned on the RicA 3D model. However, it is predictable that substitutions involving very similar residues, within the interface region, would not impair RicA-Rab2 interaction. The mapping of such substitutions could thus prevent the localization of the interface on the surface of RicA. We therefore arbitrarily removed substitutions with a score >1 in the Blosum62 score matrix, since a substitution reversing charge (K-E) has a score of 1 in this matrix. The remaining “low scoring” (LS) substitutions were positioned on the surface of the RicA 3D model (Figure 2). The same procedure was applied to substitutions occurring between *B. abortus* RicA and the *C. crescentus* RicA homolog, that were also positioned on the model (Figure 2, Additional file 1: Movie S1 and Additional file 2: Movie S2). The LS substitutions are less frequent in the regions predicted to be at the interface between monomers within the trimeric structure (Figure 3, Additional file 3: Movie S3). When all such LS substitutions are indicated on the trimeric RicA model, almost all the surface of the model is covered by substitutions, except for two regions (Figure 2A, Additional file 1: Movie S1 and Additional file 2: Movie S2). The first is the most exposed β-sheet in the RicA trimeric structure (Figure 2A). This region is probably conserved because it is involved in the folding of the β-helical component of the structure. Substitutions in this region are thus suspected to interfere with folding of the protein, which is consistent with the absence of mutations in this region in clones that still allow interaction with Rab2, since unfolded proteins are very likely unable to interact with Rab2 in the Y2H assay. The second region, smaller than the first, is the IGFP loop (Figure 1 and Figure 2A). It is a loop of the β-helical component of the structure. Since it was conceivable that mutagenesis of this loop could generate loss of interaction without affecting folding, we generated a collection of mutants in this loop.

### Mutagenesis of the IGFP loop

The IGFP loop is part of a sequence of 8 amino acids in length flanked on each side by a β strand of the LβH (Figure 1). The sequence of four amino acids Ile-Gly-Phe-Pro (IGFP) is exposed to the surface of RicA in the 3D model of the trimer (Figures 1 and 2). Loop regions of the LβH-containing proteins are known to contribute most of the residues that interact with binding partners [18,19] and surface hydrophobicity has been used to identify regions of a protein surface most likely to interact with a binding ligand [20]. We therefore proposed that this exposed IGFP loop could be involved in the recognition of Rab2.

Since the Gly and Pro residues could adopt particular ϕ and ψ torsion angles and thus their substitution may affect folding of neighboring regions, mutagenesis was limited to the Ile-70 and Phe-72 residues that were replaced with random amino acids (amino acid switches from IGFP to XGXP, where X may be one of the 20 possible amino acids). The basic procedure is described in the materials and methods section. A library of XGXP mutants (around 8,000 clones) was constructed, fused to AD domain of Gal4p and assayed for interaction with DB-Rab2 using Y2H. We observed that XGXP mutagenesis resulted in loss of interaction in about 80% of the clones (inability to drive the expression of the *HIS3* and *lacZ* reporters in the Y2H assay). We sequenced alleles generating or not interaction between RicA and Rab2, and the sequence of the XGXP loop is given in Table 2. It is detectable that the amino acid (aa) composition at the first position (70) is more variable in the RicA mutants that still interact with Rab2, compared to the second position (72). Analysis of the mutated sequences shows that slight variations at both positions, e.g. in clones 10 and 11 (Table 2), where Ile to Leu substitution occurs at position 70, and Phe to Trp and His at position 72 respectively, disrupt interaction with Rab2 in the Y2H assay.

**Table 2:** Sequenced IGFP replacements that disrupted (clones 1 to 12) or not (clones 13 to 15) RicA-Rab2 interaction

**Clone n°**	**X**^**70**^**GX**^**72**^**P sequence**	**Y2H assay with Rab2**
1	YGGP	His^-^, lacZ^-^
2	WGVP	His^-^, lacZ^-^
3	TGVP	His^-^, lacZ^-^
4	RGGP	His^-^, lacZ^-^
5	AGHP	His^-^, lacZ^-^
6	VGNP	His^-^, lacZ^-^
7	LGEP	His^-^, lacZ^-^
8	SG*P	His^-^, lacZ^-^
9	AGSP	His^-^, lacZ^-^
10	LGWP	His^-^, lacZ^-^
11	LGHP	His^-^, lacZ^-^
12	AGYP	His^-^, lacZ^-^
13	SGFP	His^+^, lacZ^+^
14	VGFP	His^+^, lacZ^+^
15	KGLP	His^+^, lacZ^+^

The * indicates a stop codon. His^-^ and His^+^ refer to the phenotype generated by the *HIS3* reporter, and LacZ^-^ and LacZ^+^ to the phenotype generated by the *lacZ* reporter.

In order to test a possible alteration of RicA-Rab2 interaction using GST pulldown, we attempted to overproduce XGXP clones n°2, 3, 4, 5, 6 and 8 as His_6_-RicA fusions. Among the 6 clones tested, none were found in the soluble extract and all were detected in the insoluble pellet, while the wild type control was found exclusively in the soluble fraction. This observation strongly suggests that mutations in the IGFP loop contribute to the proper folding of His_6_-RicA, at least in *E. coli*.

## Discussion

The objective of this study was to experimentally identify and characterize protein-protein interaction site of RicA for Rab2, to provide a better understanding of the structural basis of a human small GTPase recognition by a bacterial effector protein. In the 3D model of RicA, the protein may be divided in two parts: a N-terminal LβH component and a C-terminal α helix. Within the LβH, there are three β sheets, two embedded in the trimeric structure and one exposed to the exterior. The two internal β sheets are predicted to form the interfaces between monomers and are rarely substituted (Figure 3) in mutants generated and still able to interact with Rab2, or in the *C. crescentus* homolog. However, the loops involved in the formation of the central pore of the RicA model (visible in Figure 2B and 2C) are often mutated. The residues of the C-terminal α helix in contact with the LβH component are rarely mutated, while many exposed residues of this α helix are substituted in the mutated RicA or in the *C. crescentus* homolog. These data are consistent with the proposed 3D model of RicA.

Our data suggest that mutations in the IGFP loop that impair interaction with Rab2 also generate a folding problem. This is rather surprising because the IGFP loop is not very well conserved (except for the G and P residues, see Additional file 4: Figure S1), and moreover it is exposed to the surface of the homologous proteins of known structure ( Additional file 4: Figure S2). The role of the IGFP loop is unknown but it seems to be needed for the generation of a correct tertiary or quaternary structure, since the 6 XGXP mutants unable to interact with Rab2 are found to be insoluble when expressed in *E. coli*, while the wild type RicA is soluble. This data indicates that regions necessary for folding could overlap the regions necessary for interaction between RicA and Rab2, unless the LS substitutions do not affect interaction between RicA and Rab2. Indeed, we cannot exclude that the RicA-Rab2 interaction is sufficiently stable to be resistant to point mutations, which would preclude most of the strategies targeting loss-of-interaction mutants.

**Figure 4:**
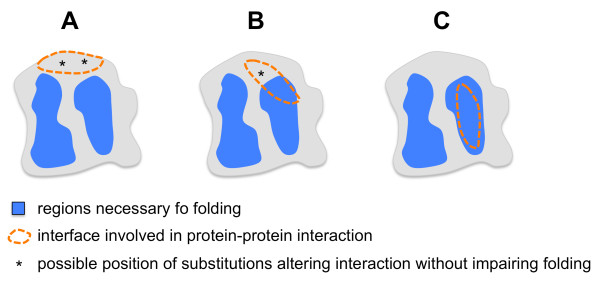
**General model illustrating the overlap between regions necessary for folding and regions required for protein-protein interaction.** If the overlap between folding and interaction regions is high, the probability to isolate substitutions impairing protein-protein interaction without preventing folding may be high (**A**), low (**B**) or very low (**C**).

To our knowledge, the identification of “edgetics” alleles (also called “interaction defective” alleles) is the easiest way to identify interaction surfaces on the structure of the proteins involved in a given interaction [16]. However, this method is only applicable if the protein to be mapped is not an autoactivator in the Y2H assay. Our example of the IGFP loop suggests that in some instances, regions necessary for folding could overlap regions involved in the protein-protein interaction (Figure 4). Such a situation could lower the probability to get “edgetics” alleles. Also, the “absence of interference” approach will generate a similar situation since the negative image produced by the absence of substitutions in a given region of the structure could simply reflect the absence of substitutions that do not affect folding of the protein. 

## Conclusion

In conclusion, we predict that for a fraction of mono-domain proteins, including *B. abortus* RicA, some structural element(s) like the IGFP loop could be bifunctional, involved in both protein folding and protein-protein interaction, as depicted in Figure 4.

## Methods

### Plasmidic constructs

*B. abortus* 2308 *ricA* coding sequence was cloned in the Gateway entry vector pDONR201 and sequenced before sub-cloning in pAD vector as a fusion protein with Gal4 activation domain (AD) and served as the prey plasmid (pAD-RicA). Human Rab2 coding sequence was cloned in pDB vector as a fusion protein with Gal4 DNA binding domain (BD) and used as the bait (pDB-Rab2). The *ricA* coding sequence was also cloned in pET15b vector (Novagen pET expression system, pET15b-RicA) as a fusion protein with N-terminal hexahistidine tag (His_6_-RicA). The *ricA* coding sequence was PCR amplified with primers that introduced N-terminal *Nde*I site and a C-terminal *Bam*HI site (*Nde*I RicA-F: 5’CAT ATG CCG ATC TAT AAC GG; *Bam*HI RicA-R: 5’GGA TCC TCA GGC AGG CTC CAT). The pET15b-RicA construct was checked by restriction diagnosis and sequencing (primer, T7: 5’TAA TAC GAC TCA CTA TAG GG).

### Generation of random mutagenesis, site directed mutagenesis and sequencing

The *ricA* mutagenesis fragment (525 bp) was synthesized by error-prone PCR [17] on the expression clone pAD-RicA with primers that hydridize to *att*B1 and *att*B2 sites that flank *ricA* in the pAD-RicA (*att*B1-F 5^′^ACA AGT TTG TAC AAA AAA GCA G-3^′^; *att*B2-R 5’ AC CAC TTT GTA CAA GAA AGC T-3^′^). Following PCR, DNA was purified and cloned into pDONR201 and the *att*L1 site primer (5^′^-CTGAAGCTTGGATCTCGGGC-3^′^) was used for sequencing. The generated random mutant library (entry clones) was sub-cloned into pAD expression vector.

Site directed mutations were incorporated into the IGFP motif using the Mutagenex^TM^ Library method. The residues Ile-70 and Phe72 were replaced with random amino acids (amino acid switches from IGFP to XGXP, where X may be one of the 20 possible amino acids). The pAD-RicA plasmid was used as template for a PCR with four synthetic oligonucleotide primers, two containing the desired mutations (FM 5^′^ ATG CAC ACC GAT NNK GGC NNK CCG CTG ACC ATC 3^′^; RM 5^′^ GAT GGT CAG CGG MNN GCC MNN ATC GGT GTG CAT 3^′^) (where N is any of A, C, G, or T; K is G or T; M is A or C) which are complementary to opposite strands of the insert, and two hybridizing to *att*B1 and *att*B2. PCR amplifications were performed for upstream and downstream regions of the mutations. A third assembly PCR was performed with *att*B1 and *att*B2 primers, using upstream and downstream PCR fragments as initial substrates. The final PCR products were cloned into pDONR201 to generate a large pool of entry-clones. We sequenced 5 randomly picked clones using the *att*L1 site primer to confirm the expected site directed mutagenesis of Ile-70 and Phe-72 codons. The entry-clone plasmidic DNA library was prepared and sub-cloned into pAD destination vector. DNA sequencing experiment was performed with the “Standard Sequencing Run" on an ABI PRISM® 3100 Genetic Analyser (Applied Biosystems). Y2H-AD (5^′^-CGC GTT TGG AAT CAC TAC AGG G 3^′^ and Y2H-Term (5^′^-GGA GAC TTG ACC AAA CCT CTG GCG 3^′^) primers were used to sequence RicA mutants still interacting with Rab2 in the Y2H.

### Y2H assays

RicA or RicA allele libraries and Rab2 were transformed into MaV203 yeast strain. MaV203 contains single copies of each three reporter genes (*HIS*3, *URA*3 and *lac*Z) that are stably integrated at different loci in the yeast genome. The interaction between RicA or its allele and Rab2 reconstituted an active transcription factor, hence the expression of reporter genes. *HIS*3 gene expression was detected by plating transformants on selective medium lacking leucine, trytophan and histidine in the presence of 3AT (20 mM). The *lac*Z reporter was tested by β-galactosidase filter assay. All controls were carried out with appropriate co-transformed vectors.

### Mapping and display of mutations on the surface of the protein

The mutations were mapped on the proposed RicA structure using MacPymol (http://www.pymol.org/). The proposed structure was obtained using EsyPred3D program [13] and verified for correctness using verify3D [14] server (http://nihserver.mbi.ucla.edu/Verify_3D/).

### Expression and purification of His_6_-RicA fusion

Purified His_6_-RicA was obtained from *E. coli* BL21 (DE3) over-expression clone. A filtered lysate was loaded into a chromatography column (Econo-Pac® cat. n° 732–1010, Biorad) pre-loaded with Ni-NTA His-Bind superflow (cat n° 70691–3, Novagen®) resin followed by wash steps and elution (45 mM Tris–HCl pH 7.9, 500 mM NaCl, 200 mM imidazole pH 7.9 and 10% glycerol).

## Abbreviations

AD: Gal4p transactivation domain; DB: Gal4p DNA binding domain; 3AT: 3-aminotriazole; LβH: Left-handed β-helical; 3D: Three-dimensional; Y2H: Yeast two-hybrid.

## Competing interests

The authors declare that they have no competing interests.

## Authors’ contributions

BN participated in the experimental design, performed all experiments unless otherwise indicated below and participated in writing the manuscript. EB tested the interaction between RicA homologs and human Rab2. JP made the gel permeation with purified RicA and tested the stability of RicA mutated in the IGFP loop. JV, JJL, JW and XDB supervised the work. All authors read and approved the final manuscript.

## Supplementary Material

Additional file 1 **Movie S1.** Is showing the 3D structure of the RicA trimer model. The color code is the same as Figures 2 and 3. The movement (120°) around the Y axis allows the distinction of zones free of substitutions (in grey). A ribbon is shown for one monomer, by transparency. Click here for file

Additional file 2 **Movie S2.** Is showing the 3D structure of the RicA trimer model. The color code is the same as Figures 2 and 3. The movement (120°) around the X axis allows the distinction of zones free of substitutions (in grey). A ribbon is shown for one monomer, by transparency. Click here for file

Additional file 3 **Movie S3.** Is showing the 3D structure of the RicA monomer model. The color code is the same as Figures 2 and 3. The movement (360°) around the Y axis allows the distinction of zones free of substitutions (in grey). The Cα trace is shown by transparency. Click here for file

Additional file 4 **Figure S1.** Is a multiple sequence alignment of RicA with 17 homologs. **Figure S2** shows the exposed loop corresponding to IGFP in RicA homologs of known structure. Click here for file
