# Supplementary material for: Structural analysis of Brucella abortus RicA substitutions that do not impair interaction with human Rab2 GTPase
Source: BMC Biochem. 2012 Aug 14;13:16. doi: 10.1186/1471-2091-13-16 (PMC3527289; doi:10.1186/1471-2091-13-16)
Supplement: Additional file 4 — Figure S1. Is a multiple sequence alignment of RicA with 17 homologs. Figure S2 shows the exposed loop corresponding to IGFP in RicA homologs of known structure. [file 1471-2091-13-16-S4.docx]

**Supplementary data**

RICAH_RHODCAPS -MIYALDGVAPVLGQG--VWVAPDANVIGKVVLEEGASVWFGCTLRGDNEEIRVGAGTNL 57

RICAH_RHODSPHA -MIYALDGVAPRIDAE--AWVAPGASVIGKVVLEAGSSVWFGAALRGDNEEIVIGAGSNV 57

RICAH_ROSDENIT MTLYALGEDIPQLHED--TWVAPGANLIGKVVLEAGASVWFGTTIRADHEEIRVGAGSNV 58

RICAH_BURKCENO MTIYKLGETAPTIHES--VFVADTAAIIGKVVLEENASVWFGATIRGDNETITVGAGSNV 58

RICA_BRUME MPIYAYNGHKPQFADRESNWIAPDATLIGKVVVGENAGFWFGAVLRGDNEPITIGADTNV 60

RICAH_OCHANT MPIYAYNGHKPLFADRSSNWIAPDATLIGKIVVGENAGFWFGAVLRGDNEPITIGDDTNV 60

RICAH_MELOTI MPLYAIDGAEPSFADAGSNWIAPDATLIGDIRVGRNAGFWFGVVIRGDNEPIIVGADTNV 60

RICAH_RHILEGUM MPVYALGGSTPKLPAAGLYWIAPDANIIGQIELGENVGVWFGAVLRGDNEKITVGEGTNI 60

RICAH_RHIETLI MPIYALGGLTPQLPPAGLHWIAPDAHVIGQVELGENVGIWFGAVLRGDNEKIAIGDGTNI 60

RICAH_AGROTUME MPLYRLADRVPQTPAPDRYWIAPDANVIGSVTLGEDVGIWFGATLRGDNEPISVGRGTNI 60

RICAH_SINOMELI MPLYALGPLRPQTPTEGSYWVAPDANIIGQVELGEDVGIWFGAMLRGDNEPIRIGARTNI 60

RICAH_METHYNOD MPLYALDDHRPRLADPARFWIAPDAHVIGQVEIGLDVNIWFTAVLRGDNEPIRLGARTNI 60

RICAH_ACIDCIT MALYELDGISPRVADS--AWVAGSAEVMGNVVLGEDASIWFGAVLRGDNETLTIGAGSNV 58

RICAH_BRADJAP MAIYELDGQAPDLPADGNYFIAETATVIGRVRLKPGASVWFGAVLRGDNEWIEIGEGANV 60

RICAH_CAUCRE MTVYSLGATTPTLPAQGEYWIAPSASVMGNVILKKNASIWWGAVARGDNDPITIGENSNV 60

RICA_HYPHNEPT MAIYEIDGVAPQLPDEGKFWIAGSAEVMGNVVLKENASVWYGCVLRGDNDPIIIGENSNI 60

RICAH_PSEUFLUO -MKHRLGDARVETHPQS--WVAPNATLVGKVRLEEGANVWFNAVLRGDNELILIGKNSNV 57

RICAH_HYPHOMIC MPLYTLDGQGVVTPPIGSFWVAPNAVLLGKVKLEEESSVWFGAVLRGDNELITVGARSNV 60

: ::* * ::* : : ..*: *.*:: : :* :*:

RICAH_RHODCAPS QESVICHTDMGFPLLIGAHCTIGHRAMLHGCTIGEGSLIGMGAVVLNGAKIGRGCLIGAG 117

RICAH_RHODSPHA QENAVLHTDMGYPLTVGANCTIGHKAMLHGCTIGENSLIGMGATVLNGAKIGRFCLIGAC 117

RICAH_ROSDENIT QENCVFHIDAGYPLRIGAGCTIGHKVMLHGCTIGDNSLIGMGATVLNGARIGANCLIGAG 118

RICAH_BURKCENO QEGAVLHTDPGFPLTIAENVTIGHQAMLHGCTIGEGSLVGIQAVVLNGAVIGRNCLVGAG 118

RICA_BRUME QEQTIMHTDIGFPLTIGAGCTIGHRAILHGCTIGENTLIGMGAIVLNGAKVGKNCLIGAG 120

RICAH_OCHANT QEHTIMHTDIGFPLTVGAGCTIGHRAILHGCTVGDNTLIGMGAIVLNGAKIGKNCLIGAG 120

RICAH_MELOTI QEHTVMHTDPGFPLTIGEGCTIGHRAMLHGCTIGDNSLIGMGAIVLNGARIGKNSLVGAG 120

RICAH_RHILEGUM QEGVMAHTDMGFPLTTGKGCTIGHHAILHGCTLGENVLIGMGATILNGAKIGSNCLVGAN 120

RICAH_RHIETLI QEGVMAHTDMGFPLTTGKGCTVGHHAILHGCTLGDNVLVGMGATILNGAKIGNNCLVGAN 120

RICAH_AGROTUME QEGVMVHSDPGFAAVIGDMCTIGHHAIVHGCSIGDNSLIGMGATILNGAKIGHNCLVGAN 120

RICAH_SINOMELI QEAVIIHVDPGHPVSIGEGCTIGHRAIVHGCTIGDNSLIGMGATILNGAKIGRNCLVGAN 120

RICAH_METHYNOD QDGAMLHTDPGFPLDLGEDVTIGHHAIVHGCTVGANSLVGMGATLLNGARIGRNCLVGAN 120

RICAH_ACIDCIT QDGSVLHSDFGQPLTLGERVTVGHKVVLHGCTVGDESLIGIGAVVLNGAKIGRNCLVGAG 118

RICAH_BRADJAP QDGSTCHTDLGFPLVIGKNCTVGHNVILHGCTIEEGALIGMGSIVMNGAKIGRNSIVGAG 120

RICAH_CAUCRE QDGSVLHTDLGAPLTIGANVTIGHMVMLHGCTIGDGSLIGIGSIVLNGAKIGKNCLIGAG 120

RICA_HYPHNEPT QDLTVIHTDIGAPVTIGKNVTVGHRVILHGCEIGDDTLIGMGSTILNRVKIGRNCIIGAN 120

RICAH_PSEUFLUO QDGTVMHTDMGYPLTIGTGVTIGHNAMLHGCTVGDYSLIGINAVILNGAKIGKNCIIGAN 117

RICAH_HYPHOMIC QDGSVLHTDPGFPLTIGEDCTVGHMAMLHGCTIGRGSLIGIGSIIMNGAKIGEECVIGAK 120

*: * * * . . *:** .::*** : *:*: : ::* . :* .::**

RICAH_RHODCAPS ALITEGKEIPDGSLVMGAPGKVVRELDAAARARLLRSAEHYRANAARFARGLTPV-- 172

RICAH_RHODSPHA ALVTEGKEIPDFSLVKGSPGKVVRELTEEERARLIASAEGYAANARRFRAGLTAL-- 172

RICAH_ROSDENIT ALITENKVIPDNSLVMGVPGKVVRELDAAAIEGLRNSALHYQKNMRRFKHEIRAIEI 175

RICAH_BURKCENO AVVTEGKTFPDNSLILGAPAKVVRELSAEDVARLRANAKTYVERRAHFKEQLVRIG- 174

RICA_BRUME TLVKEGMEIPDNSLVVGSPARVLRQLDDAAVEKLRASAKHYVERGHSFMRGMEPA-- 175

RICAH_OCHANT ALVTEGKEIPDNSLVVGSPARILRELDDAAVDKLRLSAEHYVEKARSFMRGLEPA-- 175

RICAH_MELOTI ALVTEGKEFPDNSLIVGSPAKAIRVLDDAAVERLRGSAAHYVANGKRFKAGLKKV-- 175

RICAH_RHILEGUM ALVTEGKEFPDNSLIVGAPARAIRQLDDAAIEAIRRSAENYIANWQRFARDLKQIG- 176

RICAH_RHIETLI ALVTEGKEFPDNSLIVGAPARVVRVLDEAAAEGIRRSAENYVKNWQRFARDLKQIG- 176

RICAH_AGROTUME ALVTEGKEFPDNSLIVGSPARAIRTLDEDTVAGIRRSAEKYIENWKRFSTDLAIIE- 176

RICAH_SINOMELI ALVTEGKEFPDNSLIVGAPAKMVRTLDDAAVEGLKRSAEHYVKNWQRYAAQFTLLD- 176

RICAH_METHYNOD ALVTEGKEFPDNSLIVGAPAKAVRSLDDKAVEGLRIAAQRYVANARRFAAGLKRVDP 177

RICAH_ACIDCIT ALVTEGKEFPDGSMILGSPAKAVRQLTPEQIEGLRKSAQVYIANARRFRAGLHRTG- 174

RICAH_BRADJAP SVITEGKEFPERSLIIGSPARVMRTLDDAQVQRMGSAAKFYVANGPRFTKGLKRIG- 176

RICAH_CAUCRE ALITEGKEIPDNSMVMGAPGKVVREIGEQHAMILQASALHYVENWKRYVRDLKIVE- 176

RICA_HYPHNEPT ALIPEGKEIPDNSLVMGAPGKVVKDVSEMQLQVIKMSAIHYVENWQRHARGMKRLG- 176

RICAH_PSEUFLUO SLIGEGKEIPDGSLVMGSPGKVVRELTEAQKKMLEASAAHYVHNSQRYARDLVEQEP 174

RICAH_HYPHOMIC TLIAEGKEIPPRSLVMGSPGKIVRELTDEEVERFGGAARRYVKNWRRYAEGLTLQD- 176

::: *. :* *:: * *.: :: : : * * . . :

**Figure S1. Alignment of RicA homologs**. The positions of the residues corresponding to the IGFP loop are underlined. The alignment was performed with ClustalW2 server (http://www.ebi.ac.uk/Tools/msa/clustalw2/). Sequences names were RICA_BRUME for gi|17987019|ref|NP_539653.1| [*Brucella melitensis* bv. 1 str. 16M], identical to the *B. abortus* RicA; RICAH_OCHANT for gi|153009263|ref|YP_001370478.1| [*Ochrobactrum anthropi* ATCC 49188]; RICAH_MELOTI for gi|13470465|ref|NP_102034.1| [*Mesorhizobium loti* MAFF303099]; RHILEGUM for gi|209549961|ref|YP_002281878.1| [*Rhizobium leguminosarum* bv. trifolii WSM2304]; RICAH_SINOMELI for gi|359501029|gb|EHK73660.1| [*Sinorhizobium meliloti* CCNWSX0020]; RICAH_RHIETLI for gi|190892426|ref|YP_001978968.1| [*Rhizobium etli* CIAT 652]; RICAH_METHYNOD for gi|220921627|ref|YP_002496928.1| [*Methylobacterium nodulans* ORS 2060]; RICAH_RHODCAPS for gi|294677383|ref|YP_003577998.1| [*Rhodobacter capsulatus* SB 1003]; RICAH_AGROTUME for gi|159184795|ref|NP_354523.2| [*Agrobacterium tumefaciens* str. C58]; RICAH_CAUCRE for gi|16126878|ref|NP_421442.1| [*Caulobacter crescentus* CB15]; RICAH_RHODSPHA for gi|77463475|ref|YP_352979.1| [*Rhodobacter sphaeroides* 2.4.1]; RICAH_ACIDCIT for gi|120610005|ref|YP_969683.1| [*Acidovorax citrulli* AAC00-1]; RICAH_PSEUFLUO for gi|378952602|ref|YP_005210090.1| [*Pseudomonas fluorescens* F113]; RICA_HYPHNEPT for gi|114798682|ref|YP_761350.1| [*Hyphomonas neptunium* ATCC 15444]; RICAH_ROSDENIT for gi|110679866|ref|YP_682873.1| [*Roseobacter denitrificans* OCh 114]; RICAH_HYPHOMIC for gi|338738938|ref|YP_004675900.1| [*Hyphomicrobium* sp. MC1]; RICAH_BURKCENO for gi|107028717|ref|YP_625812.1| [*Burkholderia cenocepacia* AU 1054]; RICAH_BRADJAP for gi|27380692|ref|NP_772221.1| [*Bradyrhizobium japonicum* USDA 110]


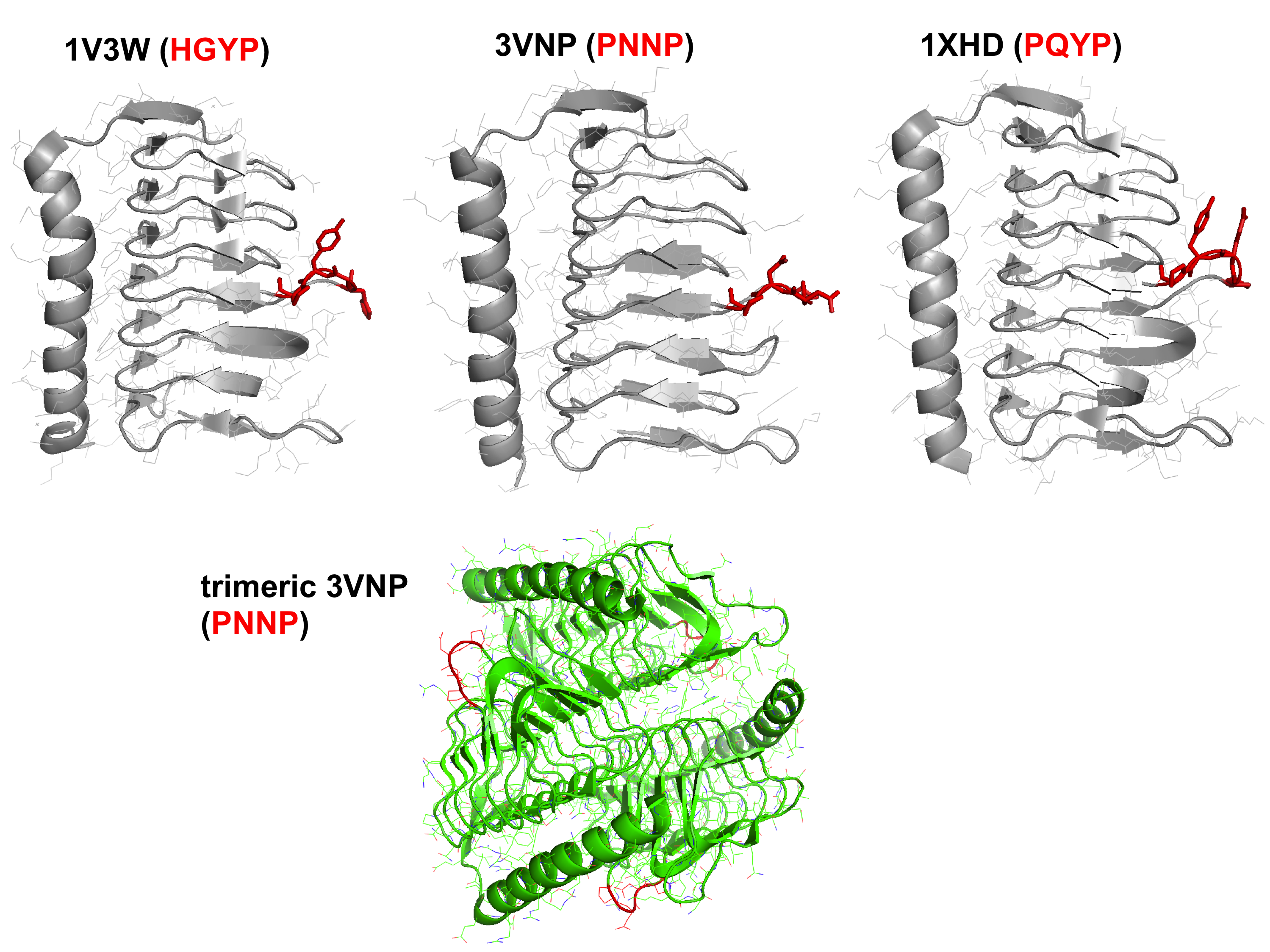


**Figure S2. Position of the loop corresponding to IGFP in RicA.** The *B. abortus* RicA sequence was used to generate a position-specific scoring matrix (PSSM) with PSI-Blast against the non-redundant database. This PSSM was used to find homologs of known structure in the Protein Data Bank. The IGFP loop was aligned to exposed loop in all three homologs of known structure, with the sequence HGYP in 1V3W, PNNP in 3VNP and PQYP in 1XHD instead of IGFP in RicA. This loop was also exposed in trimeric structures (3VNP is shown as an example).
